# Supplementary material for: Paramagnetically driven superconducting re-entrance in Eu-doped infinite layer nickelates
Source: Nat Commun. 2026 Jun 19;17:7738. doi: 10.1038/s41467-026-74065-1 (PMC13434403; doi:10.1038/s41467-026-74065-1)
Supplement: Supplementary file 1 [file 41467_2026_74065_MOESM1_ESM.pdf]

Paramagnetically driven superconducting re-entrance in Eu-doped infinite layer nickelates – Supplementary Information

Lucia Varbaro[1], Lukas Korosec[1], Chih-Ying Hsu[1,2], Pau Torruella[2], Clémentine Thibault[1], Benjamin Piot[3], David Le Boeuf[3], Duncan T.L. Alexander[2], Javier Herrero Martin[4], Weibin Li[4], Evgenios Stylianidis[5], Marta Gibert[5], Marc Gabay[6] and Jean-Marc Triscone[1]

[1]DQMP, University of Geneva, Geneva, Switzerland
[2]LSME, Ecole Polytechnique fédérale de Lausanne (EPFL), Lausanne, Switzerland
[3] Laboratoire National des Champs Magnétiques Intenses (LNCMI-EMFL), CNRS, Université Grenoble Alpes, UPS,9 INSA, 38042 Grenoble, France
[4]ALBA Synchrotron Light Source, Cerdanyola del Vallès 08290, Spain
[5] Institute of Solid State Physics, TU Wien, 1040 Vienna, Austria
[6]Laboratoire de Physique des Solides, Université Paris-Saclay, CNRS UMR 8502, Orsay, France

Supplementary Figure 1 – Scanning transmission electron microscopy (STEM) study performed on an optimally doped film of (Nd,Eu)NiO_x grown on LSAT

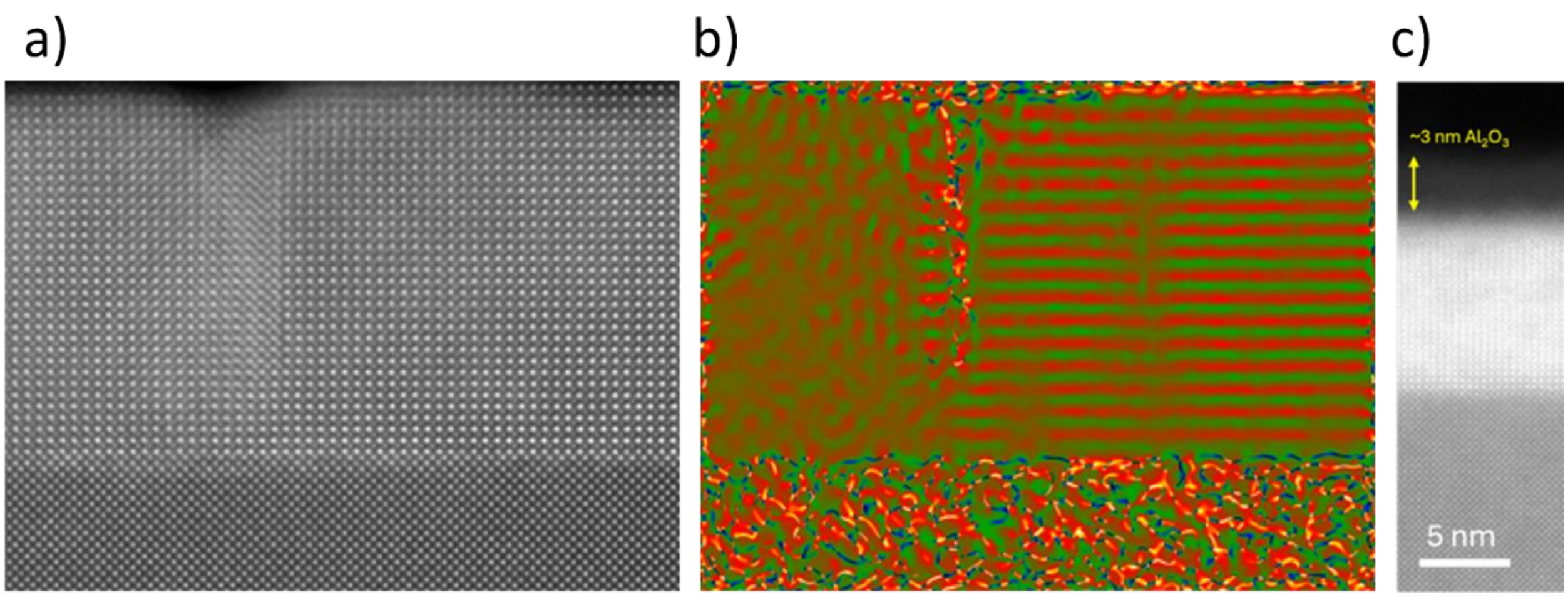

Supplementary Figure 1: (a) Cross-section STEM image of a perovskite film grown on a LSAT substrate. LSAT being cubic, the orthorhombic (Nd,Eu)NiO_3 nickelate film displays domains with different c-axis orientation (long orthorhombic axis with in-phase oxygen octahedra rotations). In (b), which presents adapted Geometrical Phase Analysis (GPA), the right hand side of the image reveals an orthorhombic domain with the long-axis pointing out of plane whose signature in GPA is horizontal fringes (for details on the technique see APL Mater. 12, 031124 (2024)). On the left hand side of the image, no fringes can be detected indicating an orthorhombic domain with the long-axis pointing in the plane of the substrate (parallel to the zone axis). (c) Low magnification image of a $Nd_{0.7}Eu_{0.3}NiO_2$ film showing the presence of the 3 nm thick Al_2O_3 capping layer right after the reduction process.

Supplementary Figure 2 – Scanning transmission electron microscopy study performed on an optimally doped film of (Nd,Eu)NiO_2 grown on LSAT

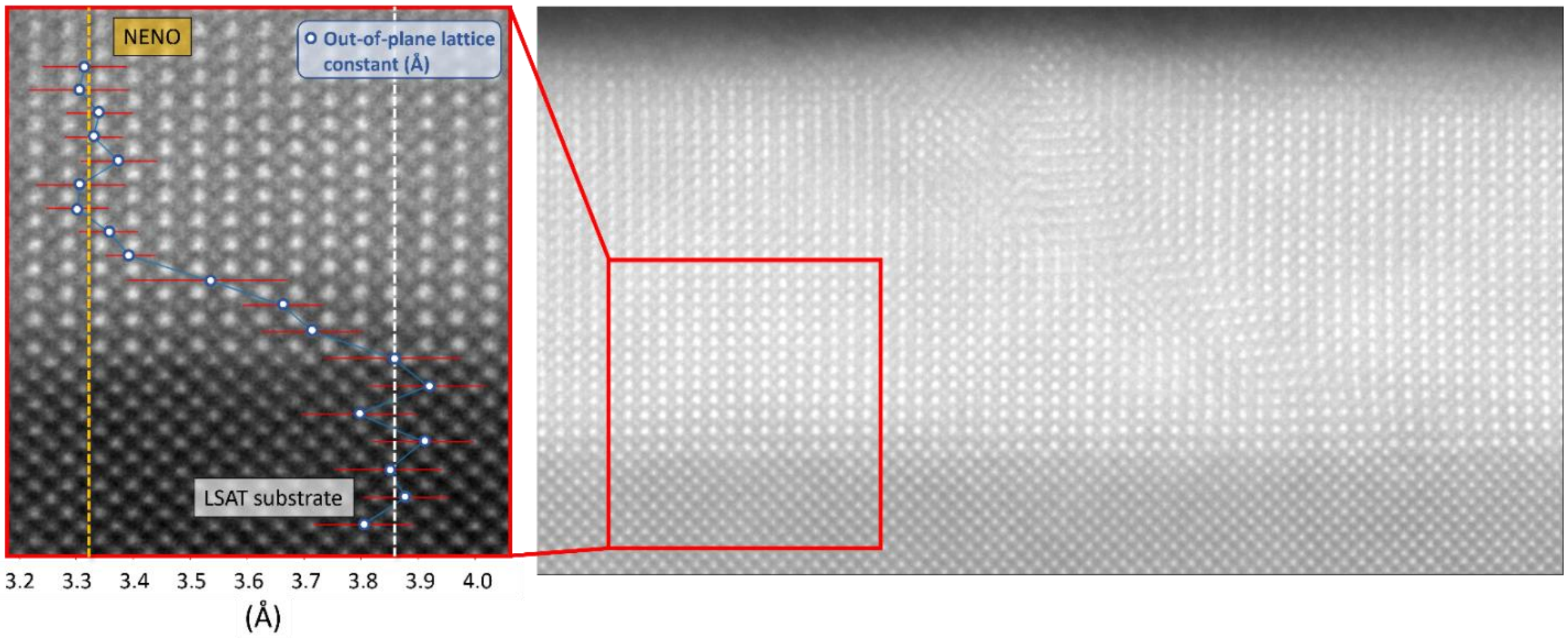

Supplementary Figure 2: Right: cross-sectional STEM image of the entire film on a LSAT substrate. The total thickness is in good agreement with that extracted from XRD fitting. The STEM image reveals the presence of a structural defect characterized by a discontinuity in the lattice periodicity, suggesting the coexistence of two distinct crystalline domains. This domain boundary may arise due to pre-existing orthorhombic domains in the perovskite phase films grown on cubic LSAT substrates (as can be seen in Fig. S1), or from inhomogeneities introduced during the reduction process.
Left: zoomed-in region of the film/substrate interface, corresponding to the red box. The local measurement of the out-of-plane lattice spacing yields a value of approximately 3.33 Å, in excellent agreement with the value obtained from XRD.

Supplementary Figure 3 – Resistivity versus temperature for an optimally doped $Nd_{1-x}Eu_xNiO_2$ film grown on $NdGaO_3$ and x-ray diffraction pattern around the 002 film reflection.

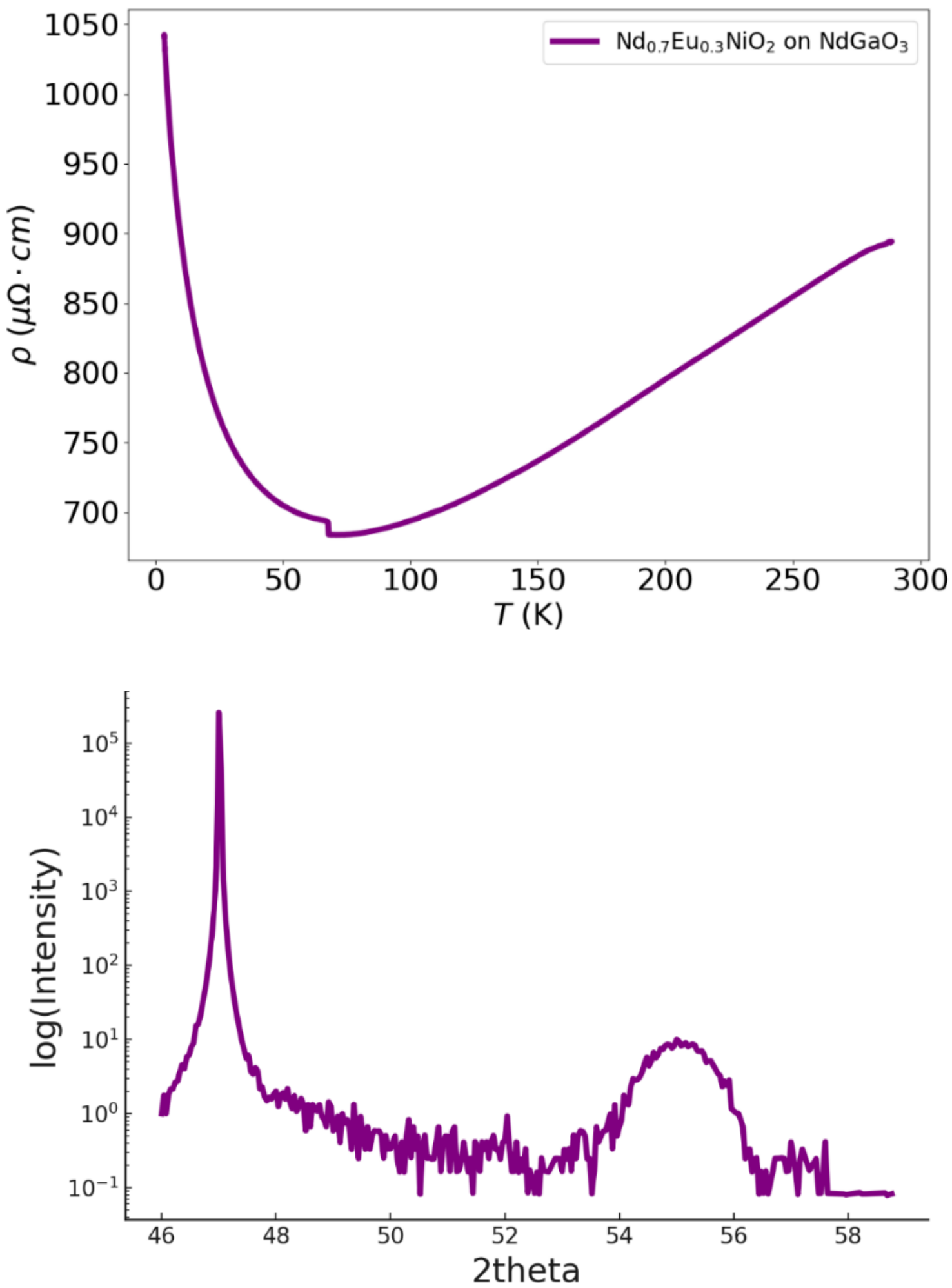

Supplementary Figure 3: Top: Resistivity versus temperature for an optimally doped non-superconducting $Nd_{1-x}Eu_xNiO_2$ sample grown on $NdGaO_3$. The sample displays a semiconducting behavior with a pronounced upturn below 70 K. The kink present at around 70 K is a measurement artefact. Bottom: X-ray diffraction scan of a sample grown on $NdGaO_3$ showing a diffraction peak around 55° (corresponding to the expected c-axis value of the infinite layer phase) and finite size oscillations, indicating a uniform, well-defined thickness and high crystalline quality, comparable to the one of samples grown on LSAT.

Supplementary Figure 4 – Temperature dependent V-I curves for different magnetic field values for the optimally doped superconducting sample

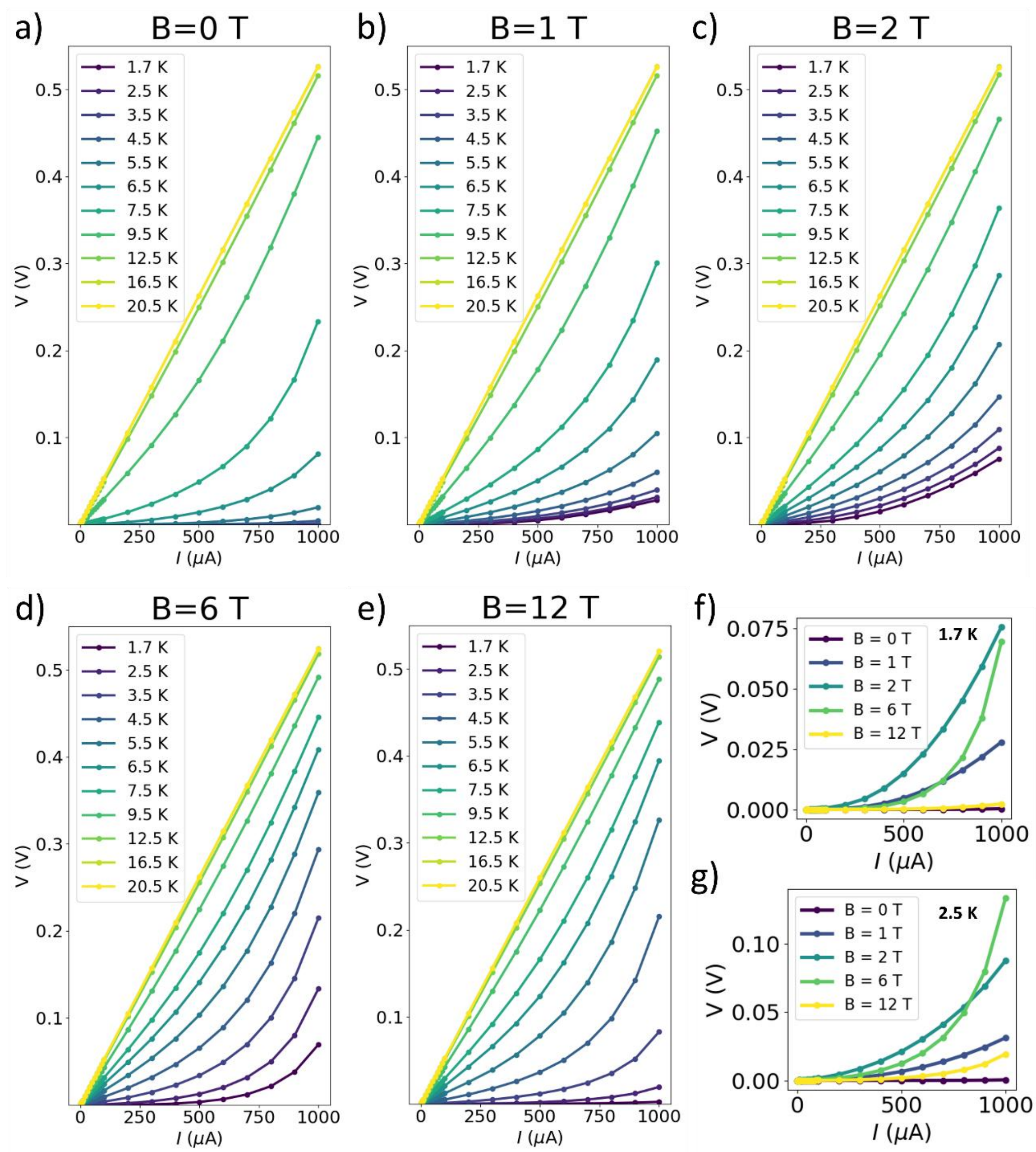

Supplementary Figure 4: Voltage-current (V-I) characteristics of an optimized $Nd_{0.7}Eu_{0.3}NiO_2$ film on LSAT measured at various temperatures under out-of-plane magnetic fields of 0 T, 1 T, 2 T, 6 T, and 12 T (a,b,c,d and e panels). At low temperatures and zero field, the film exhibits a dissipation-less state over the full current range. As the field is increased to 1 T and 2 T, the critical current is progressively suppressed, and finite voltage develops at lower current values. At higher fields (6 T and 12 T), the superconducting response reappears, with critical current magnitudes comparable to those at 0 T. These measurements confirm the existence of a re-entrant superconducting regime, consistent with the magnetic phase diagram in Figure 3 of the main text. Panels (f) and (g) show V-I curves sorted by temperature, respectively 1.7 K and 2.5 K, showing how the 0 T and 12 T critical currents are rather similar, suggesting similar superconducting state.

Supplementary Figure 5 – Electron energy loss spectroscopy on oxygen, nickel, neodymium and europium edges of an optimally doped $Nd_{0.7}Eu_{0.3}NiO_2$ film

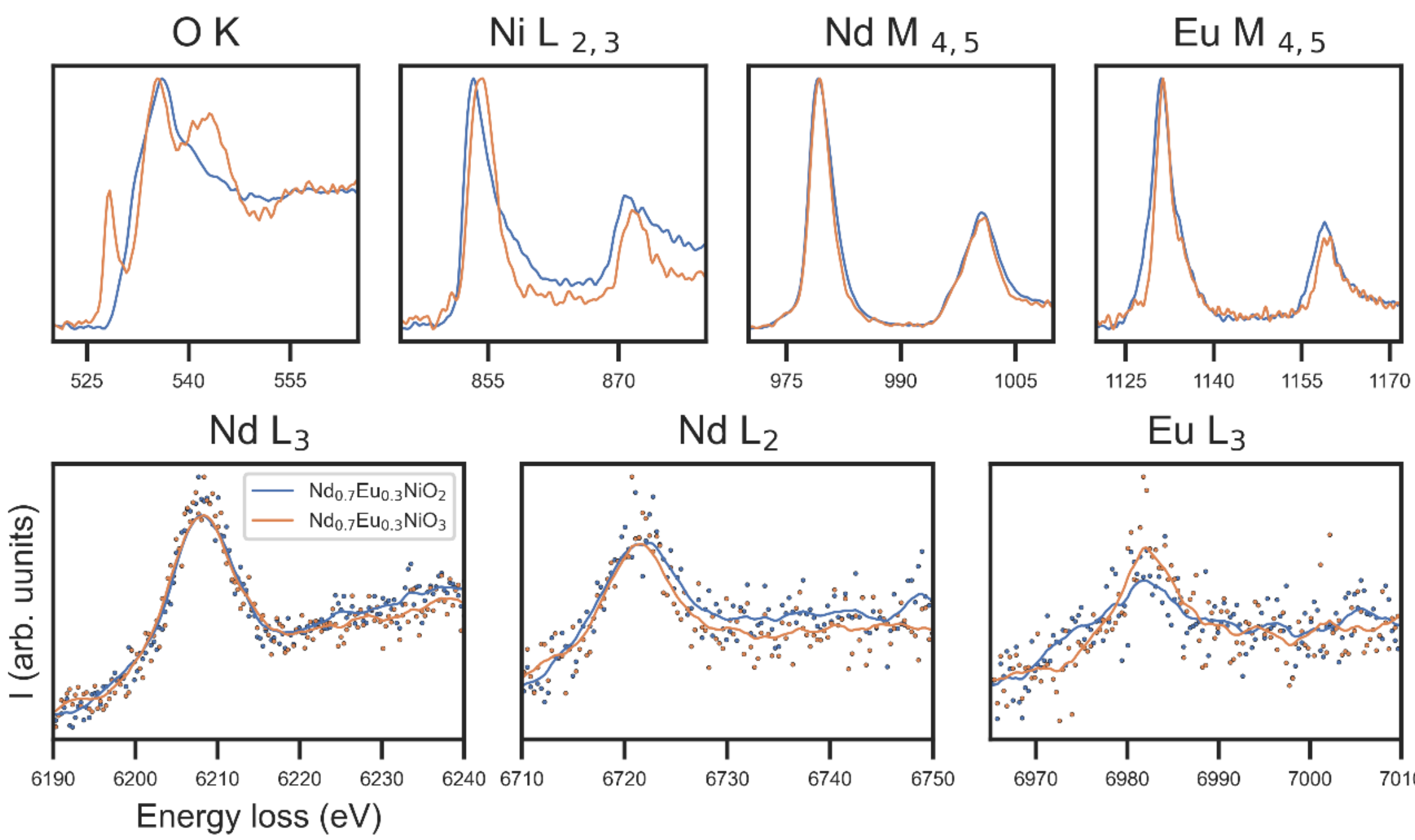

Supplementary Figure 5: STEM electron energy-loss spectroscopy (EELS) measurements for infinite layer $Nd_{0.7}Eu_{0.3}NiO_2$ and unreduced $Nd_{0.7}Eu_{0.3}NiO_3$ films on LSAT. All measurements were performed using a FEI (Thermo Fisher Scientific) Titan Themis 60-300 operated at 300 kV. The microscope is equipped with a Gatan GIF Continuum EELS spectrometer with a K3 direct electron detection camera.

For measuring the O K, Ni $L_{2,3}$, Nd $M_{4,5}$, and Eu $M_{4,5}$ edges in the top row, the beam current was set to 80 pA and a spectral dispersion of 0.18 eV/ch was applied. For these edges, a dual EELS acquisition was performed, such that the core-loss spectra could be corrected for energy shifts (by zero-loss peak alignment) and multiple scattering (by Fourier-ratio deconvolution). To improve signal-to-noise ratio (SNR), the spectra shown represent the summation of integrated, deconvolved signals from 3 separate spectrum images (SIs). Acquisition conditions were chosen to avoid beam damage effects (8 Å pixel size, subpixel scanning, 50 ms per pixel). The displayed spectra are normalized to have the same maximum intensity. Comparing the spectra of $Nd_{0.7}Eu_{0.3}NiO_3$ to those obtained from $Nd_{0.7}Eu_{0.3}NiO_2$, several clear differences appear. First, the O K edge pre-peak completely disappears in $Nd_{0.7}Eu_{0.3}NiO_2$, as observed for other doped $NdNiO_2$ infinite layer thin films, and the Ni L_3/L_2 peaks show characteristic changes in shape and intensity ratio for going from a perovskite to an infinite layer nickelate (Goodge et al., PNAS 118 e2007683118 (2021)). We note that, with this change, the Ni L edge shifts to lower energy, as can be expected for a reduction in its valence state. At the same time, going from perovskite to infinite layer, the Eu M_5 peak shifts to lower energies, and the ratio of Eu M_5 to Eu M_4 decreases, changes that both indicate a reduction of Eu^{3+} in the perovskite towards Eu^{2+} in the infinite layer compound (Mundy et al., Appl. Phys. Lett. 104, 091601 (2014)).

The lower row shows Nd and Eu L edges from the deep core-loss. Because of technical limitations, these spectra were recorded in single EELS mode, using the Nd L_3 peak to align energy offsets within/between separate SI acquisitions. At these higher energy losses, the EELS signal is much weaker. To compensate for this, multiframe acquisition (20-35 passes per SI) was used and multiple SIs were acquired, aligned and summed to improve the SNR. Sample specific acquisition parameters were carefully tuned to avoid beam damage effects (in particular,

2 nm pixel size, 50 ms per pixel, 80 pA beam current and 20 frames per SI were used for the infinite layer sample, which was the most beam sensitive one). To facilitate spectral comparison in this noisy regime, the raw data for Nd $L_{2,3}$ and Eu L_3 edges are plotted (dots), together with curves obtained by applying penalized least squares curve smoothing to the raw data (Eilers et al., Analytical Chemistry, 75, 3631 (2003)). Going from perovskite to infinite layer compound, the Eu L_3 edge splits, with a second peak appearing as a shoulder at a lower energy loss. This change points towards a reduction of Eu^{3+} towards Eu^{2+} when going from $Nd_{0.7}Eu_{0.3}NiO_3$ to $Nd_{0.7}Eu_{0.3}NiO_2$ (Wei et al., Sci. Adv. 9, eadh3327 (2023)), confirming the tendency found with the Eu M edge analysis.

Supplementary Figure 6 – Hall effect data - Temperature dependence of the transverse resistance $R_{xy}(B)$ maximum

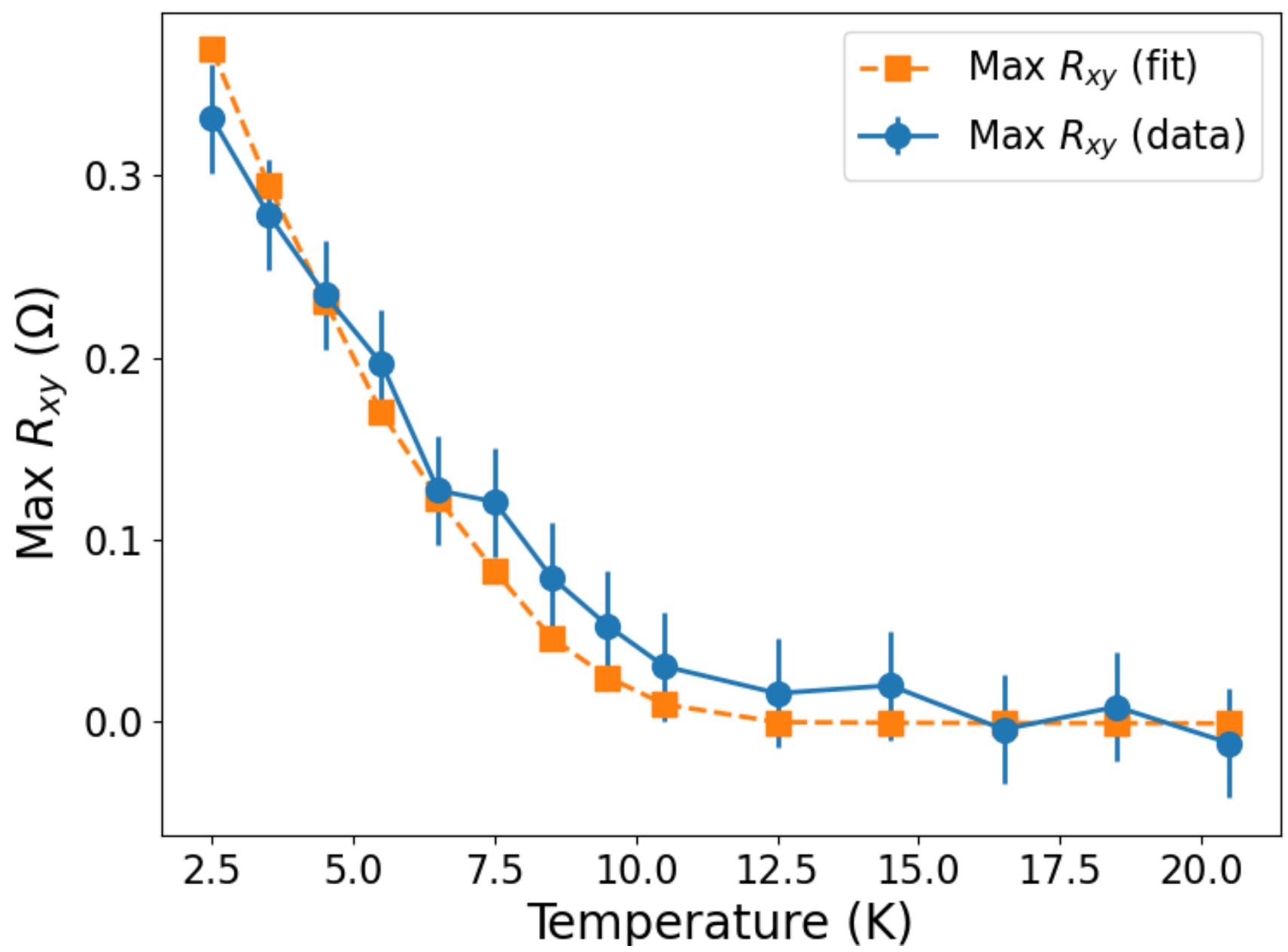

Supplementary Figure 6: Maximum of the $R_{xy}(B)$ peak - in the 0–6 T range - as a function of temperature. The experimental data (blue) are compared to the fitted model (orange dashed line), showing a good agreement and a systematic suppression of the effect with increasing temperature. The amplitude goes to zero above ~15 K implying an extremely weak polarization of the magnetic moments above this temperature that nicely links to the disappearance of the reentrant superconducting behavior above typically 10K-15K. The error bars on the experimental values were estimated from the noise level of the corresponding $R_{xy}(B)$ traces, taken as the typical amplitude of the fluctuations away from the peak.

Supplementary Figure 7 – Magnetic field and temperature dependence of the spin polarization of the Nd^{3+} and Eu^{2+} ions

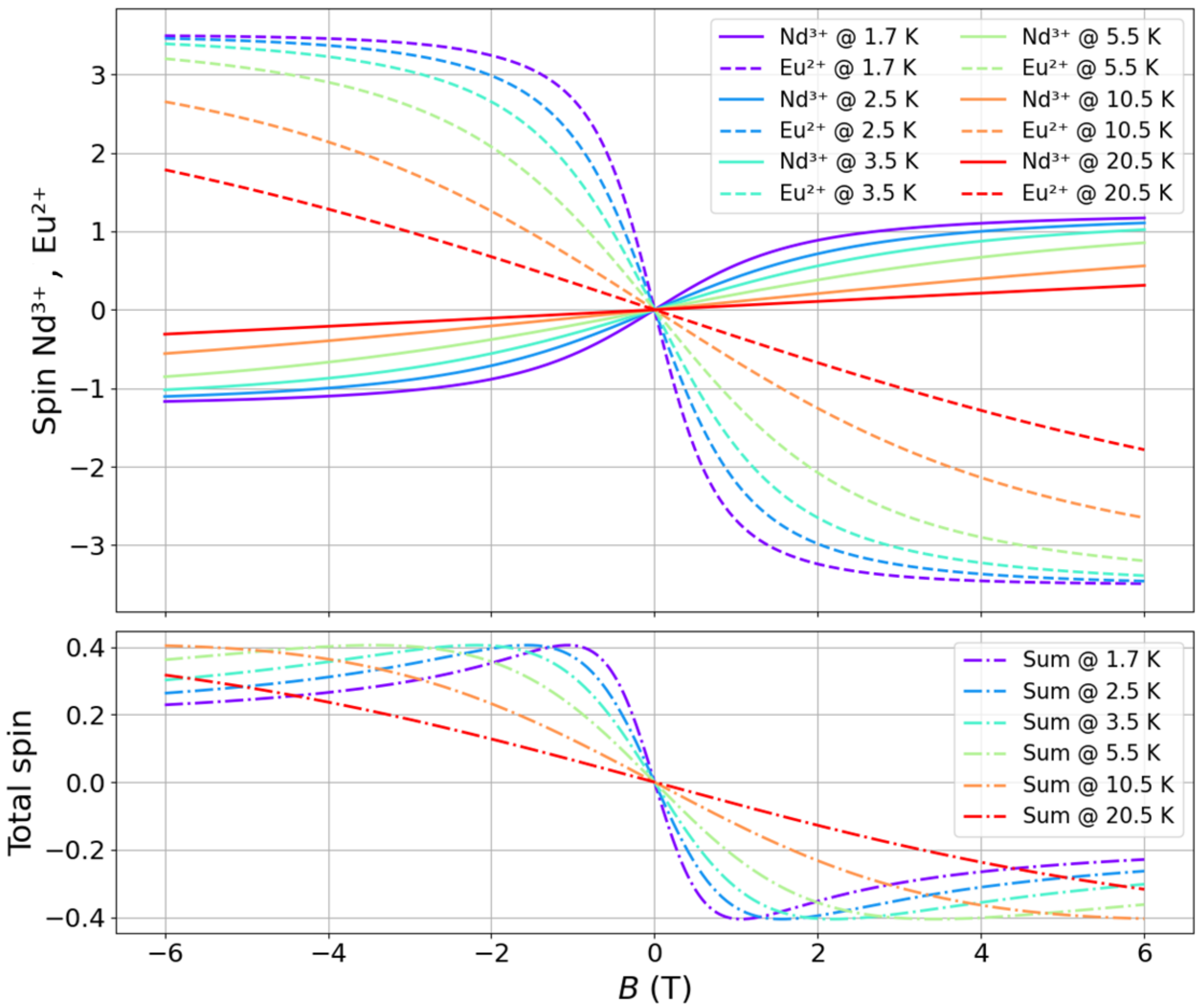

Supplementary Figure 7: Magnetic field dependence of the spin polarization of Nd^{3+} and Eu^{2+} ions calculated using the Brillouin function $B_J(x)$. Top panel, $\langle S \rangle = -(g-1)J \cdot B_J(x)$ is shown for selected temperatures between 2.5 K and 20.5 K. The solid lines represent the Nd^{3+} contribution, while dashed lines correspond to Eu^{2+}. As expected, the saturation values of the spin are 3.5 for Eu^{2+} and 1.23 (rather than 1.5) for Nd^{3+} because of the projection onto the lowest-energy J-multiplet. Due to its larger effective moment, Eu^{2+} aligns more readily with the applied field, producing a dominant contribution at low fields and low temperatures. In the bottom panel, the total spin signal is shown as the weighted sum (by the respective amounts of Eu^{2+} and Nd^{3+}) of the two contributions. This combined spin response enters the anomalous Hall model and illustrates how magnetic compensation can emerge from the interplay of the two rare-earth sublattices.

Supplementary Figure 8 – XAS and XMCD data on optimally doped $Nd_{0.7}Eu_{0.3}NiO_2$

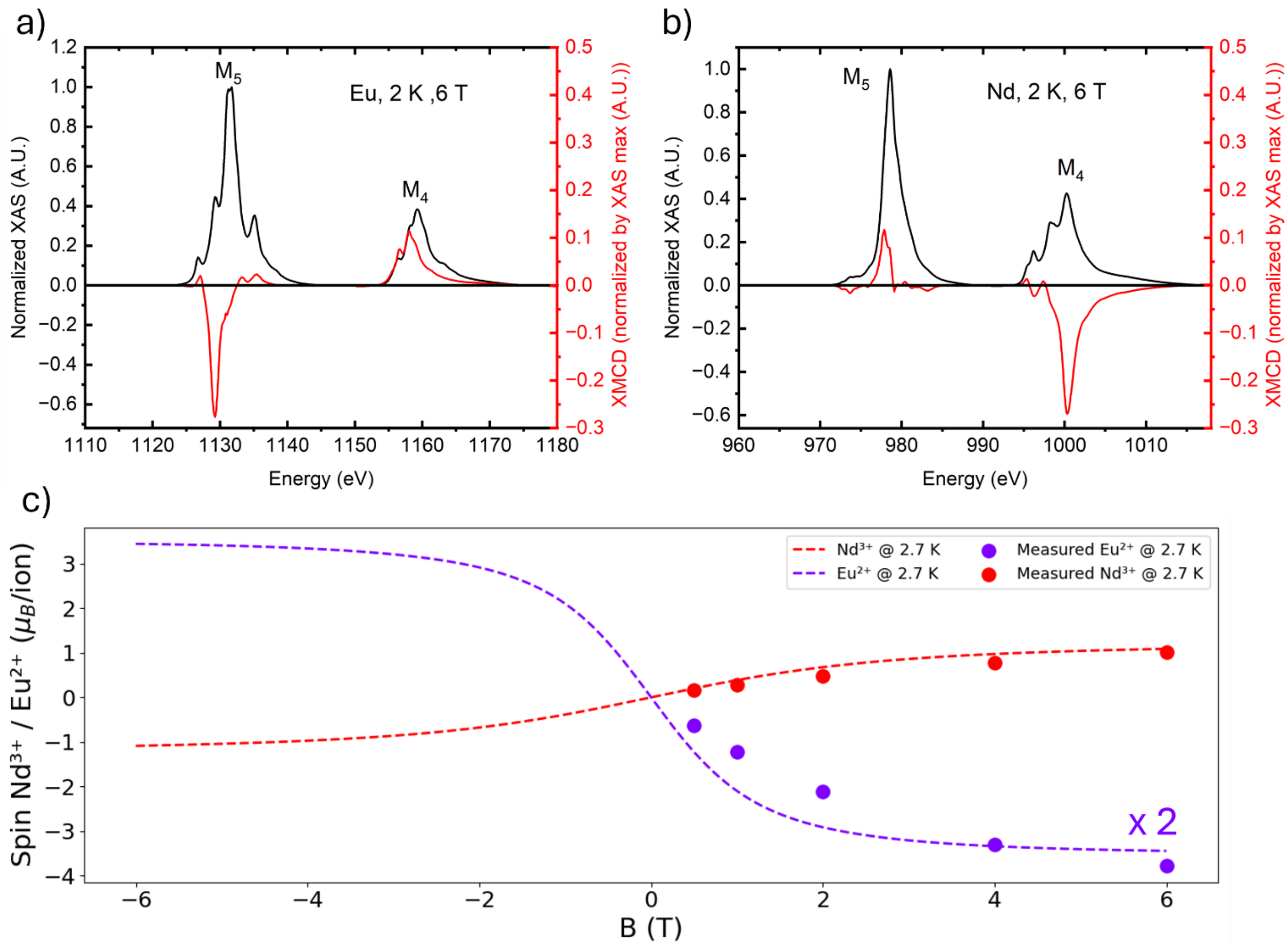

Supplementary Figure 8: (a) and (b) Normalized X-ray absorption spectroscopy (XAS) and X-ray magnetic circular dichroism (XMCD) data around the Eu and Nd $M_{5,4}$ edges, respectively, at 2 K under an applied field of 6 T. The Nd XAS data clearly indicate a Nd^{3+} state as expected. For Eu, the XAS data suggests the presence of a mixture of Eu^{2+} and Eu^{3+} states (Negusse et al., J. Appl. Phys. 99, 08E507 (2006)). We note, however, that these measurements were performed in total electron yield mode, which is surface sensitive and may therefore not fully reflect the actual situation within the bulk of the film. Nonetheless, the XMCD data around the Eu $M_{5,4}$ edges indicate that the predominant magnetic contribution arises from Eu^{2+}. (c) Comparison between the calculated spin polarizations in field and the estimated spin moments extracted from XMCD at 2.7 K by applying sum rules (Thole et al., Phys. Rev. Lett. 68, 1943 (1992), Carra et al., Phys. Rev. Lett. 70, 694 (1993)), using the method described in (Spring et al., Phys. Rev. Mater. 7, 1 (2023)). The experimental data successfully captures the antiparallel orientation of the spin moments in Eu^{2+} and Nd^{3+} and the larger spin moment of Eu^{2+}. The relatively smaller value of Eu^{2+} moment with respect to the nominal value predicted by the Brillouin function is possibly related to the presence of Eu^{3+} in the XAS spectra used to normalize the integrated XMCD signal. This might come from the overestimation of Eu^{3+} by the surface sensitive total electron yield XAS. The measured Eu^{2+} spin polarization was thus scaled up by a factor of ≈2, allowing for a comparison with the Brillouin function.

Supplementary Figure 9 – Hall effect data for a x_{Eu}=0.35 $Nd_{0.65}Eu_{0.35}NiO_2$ thin film sample

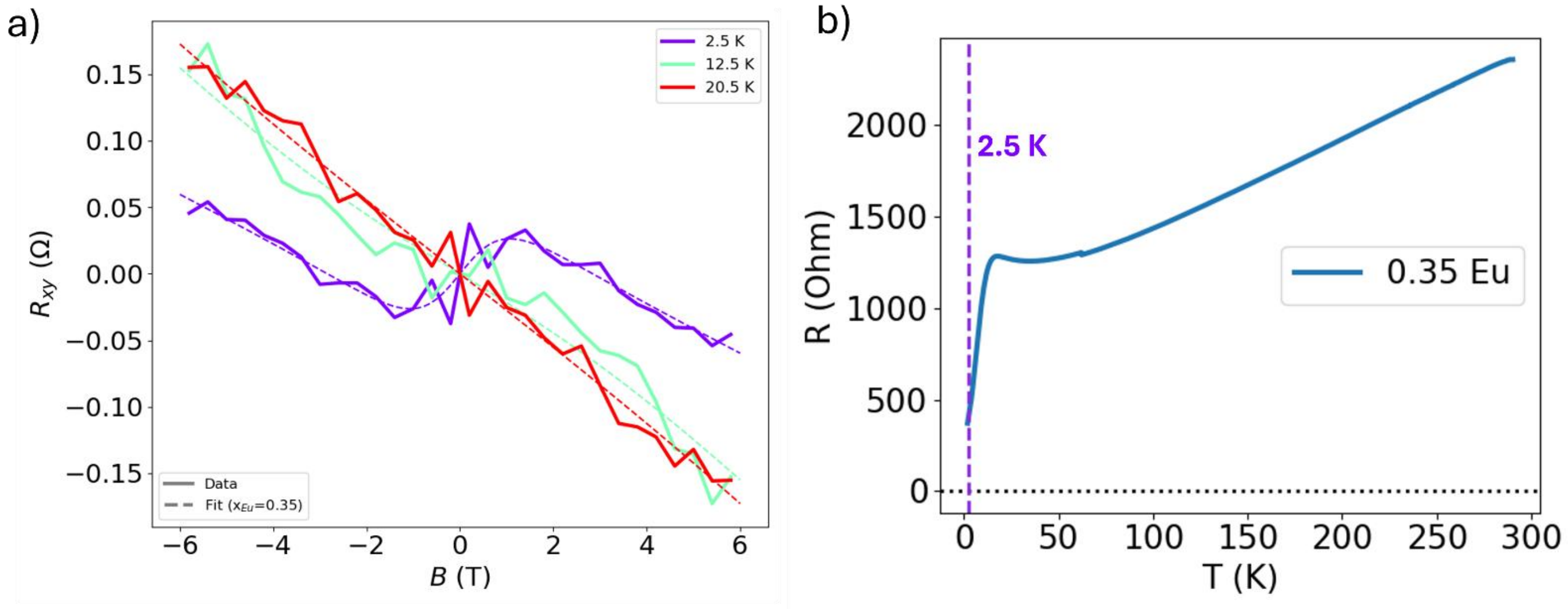

Supplementary Figure 9: (a) Hall effect data for a $Nd_{0.65}Eu_{0.35}NiO_2$ thin film grown on LSAT displaying a pronounced non-linearity, as is observed for the x=0.3 samples on LSAT and $NdGaO_3$ at low temperature (with, however, a lower amplitude due to the proximity to the zero resistance state, see dashed line in panel b) indicating the lowest temperature at which the Hall effect data was measured), that can be fitted with the same model discussed in the main text, considering a Eu concentration of 35%. (b) Resistance versus temperature data for the x=0.35 sample.

Supplementary Figure 10 – Transport properties comparison for $Nd_{1-x}Eu_xNiO_2$ samples with different compositions (x_{Eu}=0.25, 0.3)

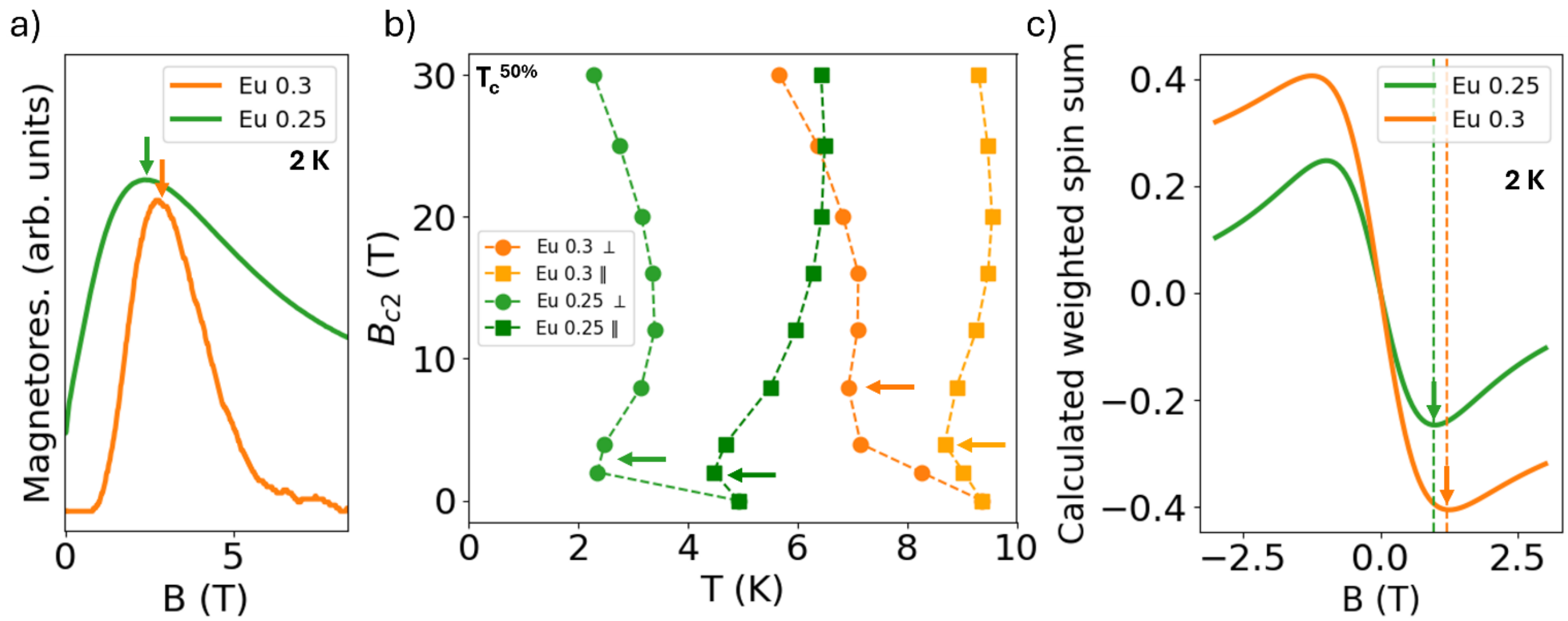

Supplementary Figure 10: a) Magnetoresistance data acquired at 2 K with an out-of-plane field configuration displaying how the dissipative state appears at lower fields when the Eu concentration is lower.

b) B_{c2} vs T phase diagram comparison in out-of-plane (circles) and in-plane (squares) magnetic field configuration for a $Nd_{0.75}Eu_{0.25}NiO_2$ and a $Nd_{0.7}Eu_{0.3}NiO_2$ thin film. The data show the same paramagnetically driven re-entrant behavior. However, with lower Eu concentrations, the minimum of T_c shifts towards lower field values, consistently with what is observed in the magnetoresistance data displayed in the left panel. For the in-plane configuration, the highest T_c is not obtained for zero field (seen particularly for the x=0.25 sample); this could perhaps signal the presence of some magnetic ordering in zero field.

c) Weighted spin sum (at 2 K) calculated through Brillouin function formalism for the two compositions, confirming the doping dependence of the magnetically induced transport properties observed in the experimental data.

Supplementary Figure 11 – Magnetic field and temperature dependence of the spin polarization of the Nd^{3+}, Sm^{3+} and Eu^{2+} ions and transport data for a $Sm_{0.35}Nd_{0.35}Eu_{0.3}NiO_2$ thin film

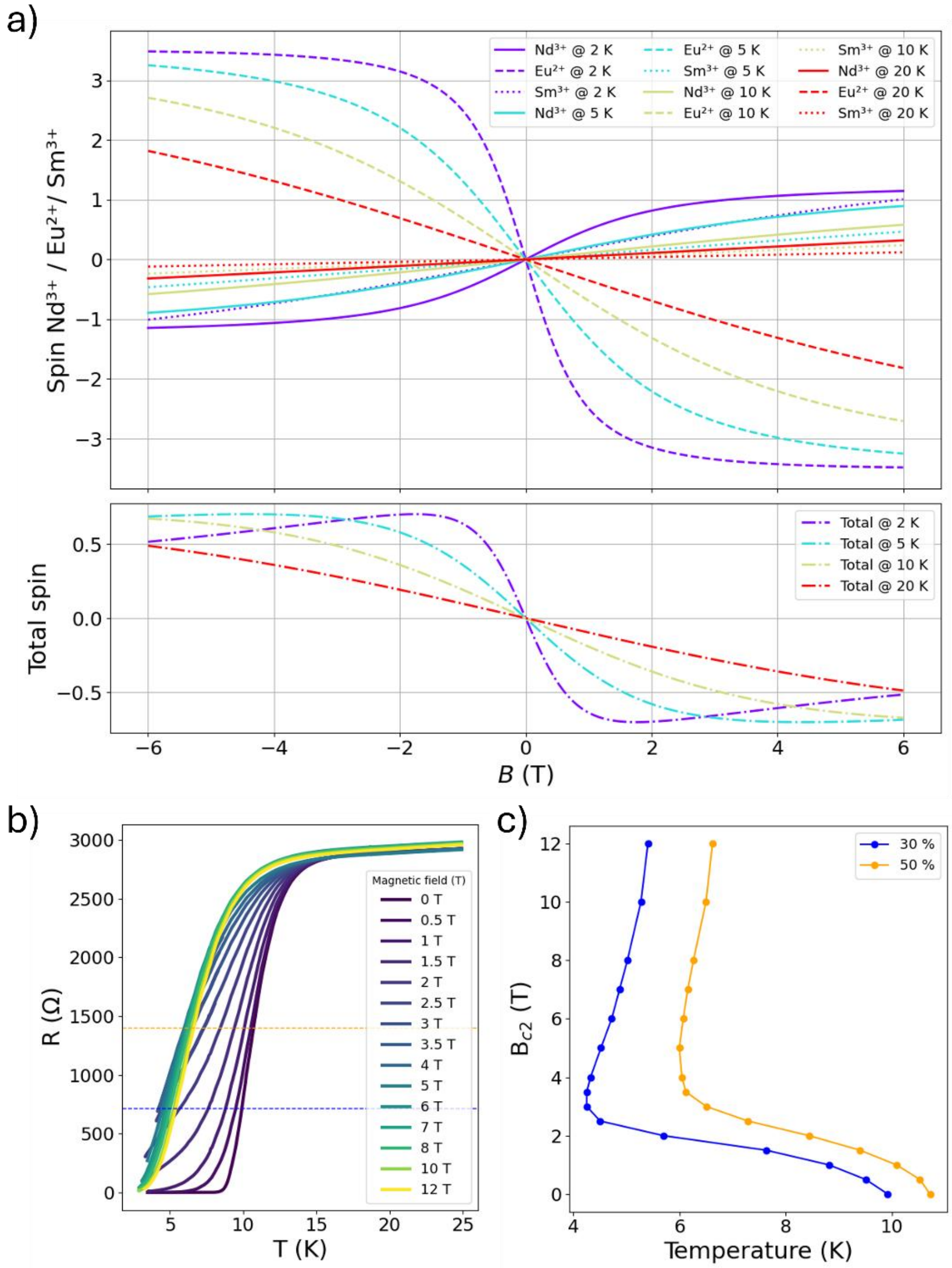

Supplementary Figure 11: (a) Calculated spin polarizations of the Nd^{3+}, Sm^{3+}, and Eu^{2+} sublattices as a function of magnetic field and temperature, using Brillouin functions with their respective g-factors and J values. While Nd^{3+} and Sm^{3+} exhibit parallel alignment, the Eu^{2+} contribution has opposite sign, resulting in a net compensation of the total spin polarization at intermediate fields (lower panel). (b) Temperature dependence of the resistance $R(T)$ measured under magnetic fields up to 12 T, showing the suppression and re-entrance of the superconducting transition.

(c) Magnetic field–temperature phase diagram extracted from the 30% and 50% R/R_N criteria, highlighting the re-entrant behavior consistent with the field range where magnetic compensation occurs.

Supplementary Figure 12 – Non-linear extraordinary Hall contributions for two $Nd_{0.7}Eu_{0.3}NiO_2$ samples grown on LSAT and $NdGaO_3$ (NGO)

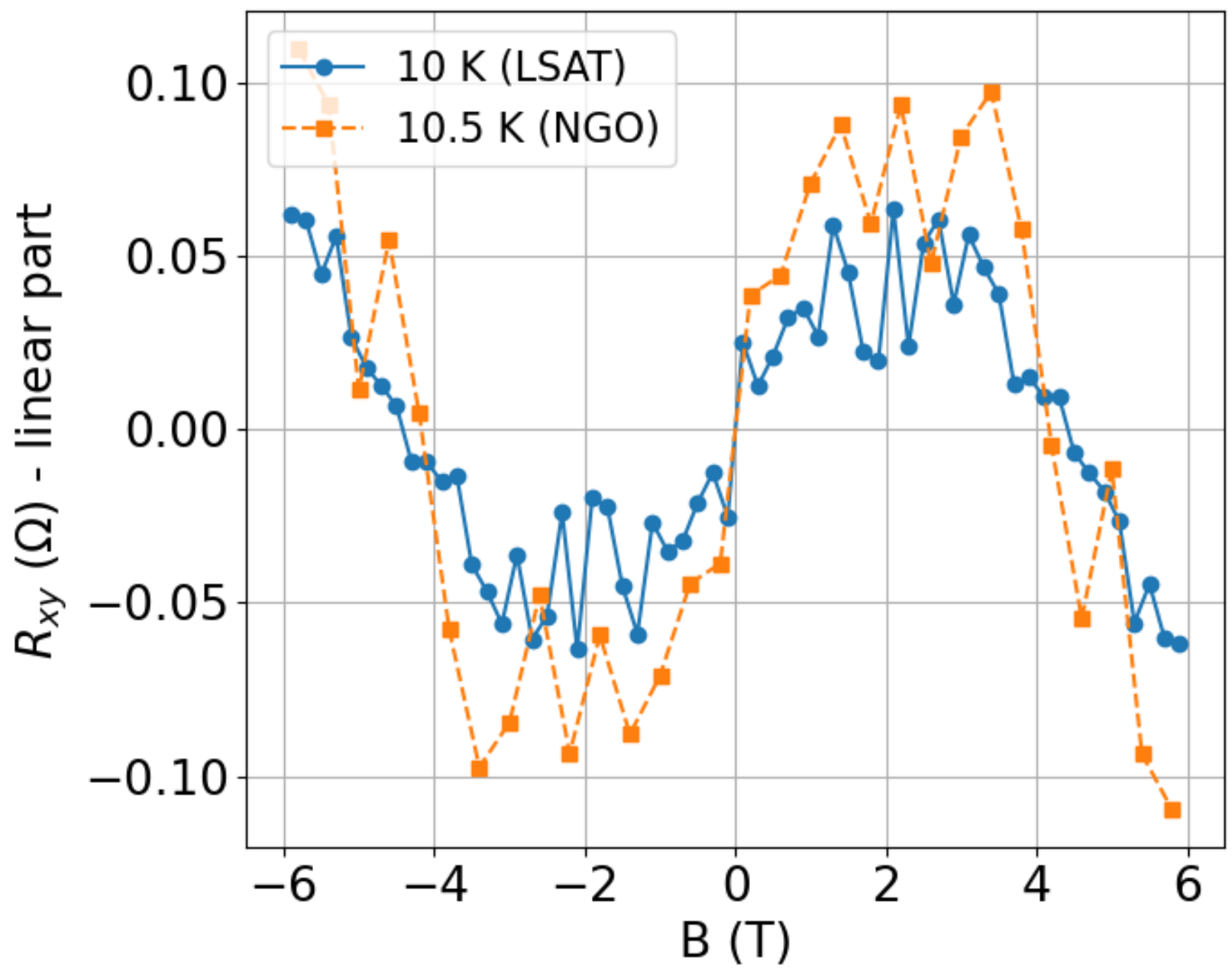

Supplementary Figure 12: Overlays of the non-linear components of the Hall resistance for a superconducting sample on LSAT (10 K, blue) with that of a non-superconducting one on $NdGaO_3$ (10.5 K, orange). The Figure shows that the non-linear extraordinary Hall contributions for the two samples are very similar in amplitude and shape.
