# Supplementary material for: Paramagnetically driven superconducting re-entrance in Eu-doped infinite layer nickelates
Source: Nat Commun. 2026 Jun 19;17:7738. doi: 10.1038/s41467-026-74065-1 (PMC13434403; doi:10.1038/s41467-026-74065-1)
Supplement: Supplementary file 2 [file 41467_2026_74065_MOESM2_ESM.pdf]

nature portfolio

Peer Review File

Paramagnetically driven superconducting re-entrance in Eu-doped infinite layer nickelates

Corresponding Author: Dr Lucia Varbaro

This manuscript has been previously reviewed at another journal. This document only contains information relating to versions considered at Nature Communications.

Version 0:

Reviewer comments:

Reviewer #1

(Remarks to the Author)
The authors have addressed all of my comments and conducted a significant amount of additional work to address the comments of all reviewers. This paper is certainly suitable for publication in Nature Communications.

Reviewer #2

(Remarks to the Author)
In the rebuttal letter and revised manuscript, the authors have thoroughly and satisfactorily addressed all of my concerns by providing additional experimental data and a more complete, in-depth discussion. Given that this is a rapidly developing topic and the results are highly interesting, I recommend the manuscript for publication in Nature Communications in its current form.

Open Access This Peer Review File is licensed under a Creative Commons Attribution 4.0 International License, which permits use, sharing, adaptation, distribution and reproduction in any medium or format, as long as you give appropriate

credit to the original author(s) and the source, provide a link to the Creative Commons license, and indicate if changes were made.
In cases where reviewers are anonymous, credit should be given to 'Anonymous Referee' and the source.
The images or other third party material in this Peer Review File are included in the article's Creative Commons license, unless indicated otherwise in a credit line to the material. If material is not included in the article's Creative Commons license and your intended use is not permitted by statutory regulation or exceeds the permitted use, you will need to obtain permission directly from the copyright holder.
To view a copy of this license, visit https://creativecommons.org/licenses/by/4.0/
